# Association between human leukocyte antigen-DR and demylinating Guillain-Barré syndrome

**Published:** 2014-10

**Authors:** Zaki N. Hasan, Haider H. Zalzala, Hyam R. Mohammedsalih, Batool M. Mahdi, Laheeb A. Abid, Zena N. Shakir, Maithem J. Fadhel

**Affiliations:** *From the HLA Typing Research Unit, Al-Kindy College of Medicine, Baghdad University, Baghdad, Iraq*

## Abstract

**Objective:**

To find an association between human leukocyte antigen (HLA) class II DRB1, DRB3, DRB4, and DRB5 alleles frequencies in a sample of Iraqi patients with Guillain-Barré syndrome (GBS) and compare with a healthy control group.

**Methods:**

We performed a cross-sectional study consisting of 30 Iraqi Arab patients with GBS attending the Neurological Department in the Neuroscience Hospital, Baghdad, Iraq between September 2012 and June 2013. The control group comprised 42 apparently healthy volunteers. Human leukocyte antigen genotyping for HLA DRB1, DRB3, DRB4, and DRB5 was performed using the polymerase chain reaction-sequence-specific primers method. The allele frequencies were compared across both groups. Major histocompatibility complex (MHC)-class II HLA-DR genotyping and serotyping were performed by software analysis.

**Results:**

We found increased frequencies of HLA genotype DRB1*03:01 (*p*=0.0009), DRB1*07:01 (*p*=0.0015), and DRB4*01:01 (*p*<0.0001) in patients with GBS compared with healthy controls. The HLA DR6 was increased in the control group (*p*<0.0001).

**Conclusions:**

Our results suggest an association between HLA-DRB1*03:01, DRB1*07:01, DRB4*01:01, and HLA DR3, DR7 and a susceptibility to GBS.

Guillain-Barré syndrome (GBS) is an acute inflammatory polyradiculoneuropathy that is autoimmune in nature, and manifests as a rapidly evolving reflexic motor paralysis with or without sensory disturbance and autonomic system involvement.^1^ Guillain-Barré syndrome is subdivided into the most common type, which is the acute inflammatory demyelinating polyneuropathy (AIDP), and an axonal variant, which is subdivided into 2 subtypes: acute motor axonal neuropathy (AMAN), and an acute motor sensory axonal neuropathy (AMSAN).^2^-^4^ There is an additional rare third type, which is Miller Fischer syndrome that accounts for only 5% of cases. The GBS prevalence is between 0.6-4/100.000 /year,^5^ with age range from 2 months to 95 years.^6^ Most patients are between 15-50 years.^7^,^8^ The autoimmune nature of the disease is triggered by a nonself-antigen (infectious agent, vaccine) that attacks gangliosides in the axonal membrane by a molecular mimicry mechanism in axonal GBS, but in AIDP, the specific antigen or antibodies are still uncertain.^8^,^9^ There is evidence that both cellular and humeral mediated immunity participates in the immune mechanism. The most common infectious agents that participate in the molecular mimicry autoimmune response are *Campylobacter jejuni*,^10^,^11^ cytomegalovirus,^12^ Epstein barr virus,^13^ and *Mycoplasma pneumoniae*.^14^

Much of the research has focused on finding the high risk predisposition of certain people with an infection to develop GBS evidenced by a twin study, linkage disease, genetic, familial, and HLA study.^15^,^16^ The HLA antigen system is the name of the loci of genes that encode for the major histocompatibility complex (MHC) in humans. These genes reside on chromosome 6, and encode cell-surface antigen-presenting proteins, along with many other functions. The HLAs corresponding to MHC class I (A, B, and C) present peptides from inside, while HLAs corresponding to MHC class II (DP, DM, DOA, DOB, DQ, and DR) present antigens from outside of the cell to T-lymphocytes. The HLAs corresponding to MHC class III encode components of the complement system.^17^ In GBS, there is macrophage activation with circulating activated T-lymphocytes evidenced by augmented expression of histocompatibility antigens (HLA-DR), suggesting that there is an association between GBS and HLA alleles.^15^,^16^ The HLA typing in GBS was investigated in several studies worldwide suggesting various associations.^15^,^16^,^18^ The aim of the current study is to investigate any association between GBS and HLA-DR.

## Methods

We performed a cross-sectional comparative study including 30 Iraqi Arab patients with GBS and attending the Neurological Department of the Neuroscience Hospital, Baghdad, Iraq between September 2012 and June 2013. Patient selection inclusion criteria were according to the criteria described by Asbury and Cornblath 1990.^19^ Patients with only demyelinating ascending polyneuropathy were included in this study. The exclusion criteria were any patient with comorbid systemic medical diseases such as diabetes, liver, renal, other neurological diseases, and other abnormal electromyography due to other causes. The control group consisted of 42 healthy volunteers among the staff of Al-Kindy College of Medicine, Baghdad, Iraq that did not have any neurological disorders whether recent or previously, and had a negative family history for this diseases or other neurological disorders. The control group was ethnically similar to the patients group. The age of the patient group ranged from 9-72 years, and the control group ages ranged from 22-75 years. The male to female ratio was 3:2 in the patient group, and 2:1 in the control group. The Scientific Ethical Committee of Al-Kindy College of Medicine approved the study. We obtained informed consent from all patients and members of the control group.

### HLA genotyping

Peripheral venous blood samples from patients and control groups were collected in ethylene diamine tetra acetic acid-containing tubes and then stored at -20°C until testing for class II- HLA-DRB1, HLA-DRB3, HLA-DRB4, and HLA-DRB5 using the polymerase chain reaction (PCR)-sequence specific primer (SSP) method. Genomic DNA was extracted using Promega DNA extraction Kit- (Promega Corporation, Fitchburg, Wisconsin, USA). All DNA was stored at -20°C until tested. The HLA class II typing was performed using PCR-SSP according to the manufacturer instruction using a Micro SSP generic Class II DNA typing tray-DRB only with lot number of SSP2LB-004 (One Lambda, Canoga Park, California, USA). The results were interpreted by HLA fusion software version 2.0 (One Lambda, Canoga Park, California, USA). Serological results were assigned by software analysis. The HLA typing was carried out in the HLA research unit at Al-Kindy College of Medicine.

### Statistical analysis

The distribution of HLA alleles in the patient and control groups was compared using chi-square for continuous variables using MiniTab version 15 software (Minitab Inc., State College, PA, USA). Fisher’s exact test was used when necessary (cell data <5). In each comparison, the odds ratio (OR) along with the 95% confidence interval (95% CI) was used. A *p*-value less than 0.05 was considered statistically significant.

## Results

The age of patients ranged from 9-72 years, with a mean age of 30±16.11 years. The control age ranged from 14-75 years with a mean of 36±17.51. Eighteen of the patients were male compared with 28 males in the control group, with 12 females in the patient group, and 14 females in the control group. The alleles frequencies of HLA-DRB1*, DRB3*, DRB4*, and DRB5* for GBS patients and the control group are shown in **Table 1**. There was a statistically significant increased frequency of HLA-DRB4*01:01 in patients with GBS compared with healthy controls. There was also a statistically significant increase in the HLA-DRB1*03:01, and HLA-DRB1* 07:01 in patients with GBS compared with the control group (**Table 1**). **Table 2** shows the frequency of the HLA serotype alleles in GBS patients and the healthy control group. We found a statistically significant increase in the HLA DR3, and DR7 serotype in GBS patients, while the HLA DR6 serotype was statistically significantly increased in the control group. This indicates that HLA-DR6 is a protective allele against development of this disease. The frequency of HLA genotype alleles in patients who developed complications and required mechanical ventilation in comparison with those with an uncomplicated disease course where tested in our study, but failed to show a statistically significant difference (**Table 3**).

**Table 1:** Human leukocyte antigen (HLA) class II DRB genotypes in Iraqi Arab patients with Guillain-Barré Syndrome in comparison with a healthy control group.

HLA-alleles	Guillain-Barré syndrome patients group (n=30)	Healthy control group (n=42)	Odds ratio (95% confidence interval)	*P*-value
	n (%)	
DRB1*				
03:01	14 (46.7)	4 (9.5)	8.312 (2.36-29.17)	0.0009
03:17	0	5 (11.9)	NA	NA
03:42	2 (6.7)	0	NA	NA
03:76	2 (6.7)	0	NA	NA
04:01	4 (13.3)	0	NA	NA
07:01	12 (40.0)	2 (4.8)	13.33 (2.7-65.84)	0.0015
08:01	0	2 (9.1)	NA	NA
11:01	4 (13.3)	2 (4.8)	-	0.213
11:02	2 (2.6)	2 (4.8)	-	0.729
11:03	0	7 (16.7)	NA	NA
11:119	2 (6.7)	0	NA	NA
11:13	2 (6.7)	0	NA	NA
11:67	0	8 (19.0)	NA	NA
12:01	4 (13.3)	0	NA	NA
12:09	0	2 (4.8)	NA	NA
13:01	6 (20.0)	2 (4.8)	-	0.44
13:05	0	3 (7.1)	NA	NA
13:116	0	5 (11.9)	NA	NA
13:119	0	8 (19.0)	NA	NA
13:18	0	4 (9.5)	NA	NA
14:01	0	2 (4.8)	NA	NA
14:02	0	3 (7.1)	NA	NA
14:16	0	3 (7.1)	NA	NA
14:57	0	4 (9.5)	NA	NA
15:01	6 (20.0)	0	NA	NA
DRB3*				
01:01	24 (80.0)	40 (95.0)	-	0.0602
DRB4*				
01:01	18 (60.0)	2 (4.7)	30.000 (6.07-148.14)	0.0001
DRB5*				
01:01	4 (13.3)	0	NA	NA

NA - not applicable

**Table 2:** Major histocompatibility complex class II (human leukocyte antigen-DR) serotypes in Iraqi Arabic patients with Guillain-Barré syndrome in comparison with a healthy control group.

Human leukocyte antigen serology allele	No. of alleles in patients (n=30)	No. of alleles in controls (n=42)	Odds ratio (95% confidence ratio)	*P*-value
	n (%)			
DR2	6 (20.0)	0		
DR3	18 (60.0)	9 (21.4)	5.5 (1.9485-15.5249)	0.0013
DR4	4 (13.3)	0	N.A	NA
DR5	14 (46.7)	19 (54.5)		0.9054
DR6	6 (20.0)	32 (45.2)	0.0806 (0.0257-0.2531)	0.0001
DR7	12 (40.0)	2 (4.8)	13.3333 (2.7001-65.8423)	0.0015
DR8	0	2 (4.8)	NA	
DR9	0	0	NA	
DR10	0	0	NA	

**Table 3:** Major histocompatibility complex class II (human leukocyte antigen [HLA]-DR) serotypes in Iraqi Arabic patients with Guillain-Barré syndrome (GBS) in comparison with a healthy control group.

HLA alleles	GBS patients with complications (n=12)	GBS patients without complications (n=18)	*P*-value
	n (%)		
DRB1*			
03:01	6 (50.0)	8 (44.4)	1.000
03:42	0	2 (11.1)	NA
03:76	0	2 (11.1)	NA
04:01	2 (16.7)	2 (11.1)	1.000
07:01	4 (33.3)	8 (44.4)	0.708
11:01	2 (16.7)	4 (33.3)	1.000
11:02	2 (16.7)	0	NA
11:119	2 (16.7)	0	NA
11:13	0	2 (11.1)	NA
12:01	0	4 (33.3)	NA
13:01	6 (50.0)	0	NA
15:01	2 (16.7)	4 (33.3)	NA
DRB3*			
01:01	10 (83.3)	14 (77.8)	1.000
DRB4*			
01:01	6 (50.0)	12 (66.7)	0.458

## Discussion

Guillain-Barré syndrome is an autoimmune disorder. Many researchers have tried to find the cause of the disease to prevent its occurrence. One of the factors that may have an association with the disease occurrence is HLA, which plays an important role in the body’s immune responses and development of autoimmune diseases especially class II HLA-DR and DQ.^20^ Guillain-Barré syndrome and its association with HLA typing are being studied in different countries worldwide.^16^,^21^ In this study, we found a significant association between HLA-DRB4*01:01, HLA-DRB1*07:01, and HLA-DRB1*03:01 in GBS patients with a significant increase of the HLA -DR2 and DR7 serotypes. This agrees with the result of Fekih-Mrissa et al’s study^22^ who reported an increase in HLA-DR3 in Tunisian cases. However, the HLA DR6 serotype was increased in the control group, and that can be considered as a protective factor in the disease occurrence. We did not detect any increase in the frequency of HLA-DR in patients requiring mechanical ventilation, which is in contrast to Geleijns et al’s^18^ study that demonstrated an increase in the frequency of HLA-DRB1*01 in patients requiring mechanical ventilation. McCombe et al^23^ demonstrated an increase of the HLA-DR2 serotype in GBS female patients. These differences with other studies may be due to race, patients selection, religion, method used, sample size, and other environmental factors that contribute to the development of the disease, and the results indicate an aberrant genetic makeup of the patients that made them more susceptible to develop the syndrome after exposure to the environment. Although a sporadic disease could be developed, other studies have reported familial cases of GBS, which postulate a genetic susceptibility making such cases worth reporting.^24^ Few studies have assessed HLA typing in familial GBS.^21^

The limitations of this study are in the sample size and the method used in HLA typing. Regarding sample size, it is small since there are few cases of GBS, and we recommend carrying out further similar work on a larger sample size. Regarding the method of HLA typing, we used the PCR-SSP method, and we recommend using other advanced molecular methods, such as PCR-sequence specific oligonucleotide and DNA sequencing to increase the possibilities of discovering new allele polymorphisms.

In conclusion, we found an increased frequency of HLA-DRB4*01:01, DRB1*03:01, and HLA-DRB1*07:01 in patients with GBS compared with healthy controls, while HLADR6 showed an increased frequency in the control group. However, our findings differ from other studies due to significant variations in age, gender, ethnicity, and racial background.
